# Nanoparticle-based Chemiluminescence for Chiral Discrimination of Thiol-Containing Amino Acids

**DOI:** 10.1038/s41598-018-32416-z

**Published:** 2018-09-18

**Authors:** Maryam Shahrajabian, Forough Ghasemi, M. Reza Hormozi-Nezhad

**Affiliations:** 1 Department of Chemistry, Sharif University of Technology, Tehran, 11155-9516 Iran; 2 Institute for Nanoscience and Nanotechnology, Sharif University of Technology, Tehran, Iran

## Abstract

The ability to recognize the molecular chirality of enantiomers is extremely important owing to their critical role in drug development and biochemistry. Convenient discrimination of enantiomers has remained a challenge due to lack of unsophisticated methods. In this work, we have reported a simple strategy for chiral recognition of thiol-containing amino acids including penicillamine (PA), and cysteine (Cys). We have successfully designed a nanoparticle-based chemiluminescence (CL) system based on the reaction between cadmium telluride quantum dots (CdTe QDs) and the enantiomers. The different interactions of CdTe QDs with PA enantiomers or Cys enantiomers led to different CL intensities, resulting in the chiral recognition of these enantiomers. The developed method showed the ability for determination of enantiomeric excess of PA and Cys. It has also obtained an enantioselective concentration range from 1.15 to 9.2 mM for PA. To demonstrate the potential application of this method, the designed platform was applied for the quantification of PA in urine and tablet samples. For the first time, we presented a novel practical application of nanoparticle-based CL system for chiral discrimination.

## Introduction

Chiral recognition is considered as one of the most essential yet difficult measurements of molecular recognition. Chiral identification is a key process in fields of biochemistry, biotechnology, modern chemistry, biology, development of asymmetric catalysts^1^, and production of pharmaceuticals^2^. Enantiomers of chiral molecules may express extremely different effects in terms of biochemical activities, potency, mechanism, metabolic pathways^3^, stereo-specific synthesis, production of pharmaceuticals^2^, toxicity, and metabolism^1^. For instance, in biological processes, enantiomers generally exhibit different pharmacological functions and toxicities on living organisms^4^. Human body responses differently to different enantiomers of some chiral compounds and only one enantiomer may interact desirably, while the other may induce serious dangerous effects^5^.

Penicillamine (PA) is a critical chiral drug for many therapeutic applications. It exists in two isomeric forms, including D-penicillamine (D-PA) and L-penicillamine (L-PA). The important pharmaceutical form is D-PA which is used in the treatment of Wilson’s disease, rheumatoid arthritis, and hepatitis. D-PA is also applied for the prevention of infants’ retina disease in preterm infants^4^, and cystinuria^5^. It is also an important intermediate in many pharmaceutical syntheses. In addition, D-PA can be used as an antidote in some heavy-metal poisoning. In contrast, L-PA has been found to have toxic properties since it may contribute to several considerable adverse reactions like osteomyelitis and neuritis^6^. lt can also inhibit the action of pyridoxine^7^. Another thiol-containing amino acid, cysteine (Cys) has vital biological functions in neuronal tissues, metabolism, brain, detoxification, and protein synthesis^8–10^. Enantiomers of Cys express extremely different effects on fundamental physiological processes. As an example, L-Cys is essential for living beings and D-Cys is useful in tumor treatment^11^. Cys is useful for protecting normal tissues against the undesirable side effects of cancer chemotherapeutic agents and radiation treatment. Radioprotective and chemotherapeutic strategies might be developed using the D-Cys^12^. L-Cys aids many substantial cellular functions such as metabolism and detoxification. Low levels of L-Cys normally acts as a neuroprotective antioxidant in neuronal activity, while high levels of L-Cys may cause neurotoxic effects that may lead to following neuronal trauma like brain ischemia. In contrast, D-Cys does not lead to excitotoxic damage to the brain and protects cerebellar neurons from oxidative stress induced by hydrogen peroxide^8^. Thus the development of a simple method for chiral recognition of Cys and PA, and determination of their enantiomeric excess is extremely important in the fields of pharmaceutical science clinical medicine, and biochemistry.

Different analysis methods including CL^13^, biamperometry^14^, fluorometry^15–18^, high-performance liquid chromatography^19,20^, and spectrophotometry^21–24^ have been proposed for the identification of D-PA. However, these methods cannot make an effective discrimination between D- and L- enantiomers. On the other hand, various analytical techniques, including high-performance liquid chromatography (HPLC), thin-layer chromatography (TLC), capillary electrophoresis (CE), gas chromatography, mass spectrometry^25–36^, circular dichroism (CD)^37^, fluorometry^38^, nuclear magnetic resonance (NMR) protocols^39,40^ and using chiral light fields^41–44^ have been reported to distinguish the chirality of PA, Cys, and other chiral compounds. However, most of these techniques have several drawbacks such as laborious setup process, expensive chiral columns, complicated sample pretreatment, complex operation process and high-cost chiral stationary phases or chiral selectors. Therefore, the design of inexpensive and simple methods for chiral recognition of enantiomers is still a great challenge.

Intrinsic chirality of nanoparticles(NPs) and the relationship between the chirality of nanoparticles and chirality of molecules on their surface are interesting topics that have been extensively studied in the past years^45–48^. Chemical sensing based on nanoparticles has attracted research interests due to their unique size-dependent properties. The catalytic effect of NPs also leads to excellent CL signal amplification, leading to their efficient use in chemiluminescence (CL) sensors. Because of simple, inexpensive, and low background signal features, NP enhanced-CL systems can be used as efficient sensors^49–52^. There are few reports on NP-based recognition of chiral compounds^53–58^ but to the best of our knowledge, no study has so far been published on NP-based CL sensor for chiral discriminations.

As we have reported previously, thioglycolic acid (TGA) capped cadmium telluride QDs (TGA-CdTe QDs) can act as an enhancer for the luminol–H_2_O_2_ CL system to generate strong CL radiation^59^. In the present work, a new CL method based on TGA-CdTe QDs has been proposed for the discrimination of PA and Cys enantiomers. It was found that different affinity of L- and D- enantiomers of PA and Cys towards CdTe QDs leads to different CL intensities, resulting in the chiral recognition of these thiol-containing amino acids.

## Results and Discussion

### Chemiluminometric chiral recognition of PA and Cys enantiomers

**Figure 1:**
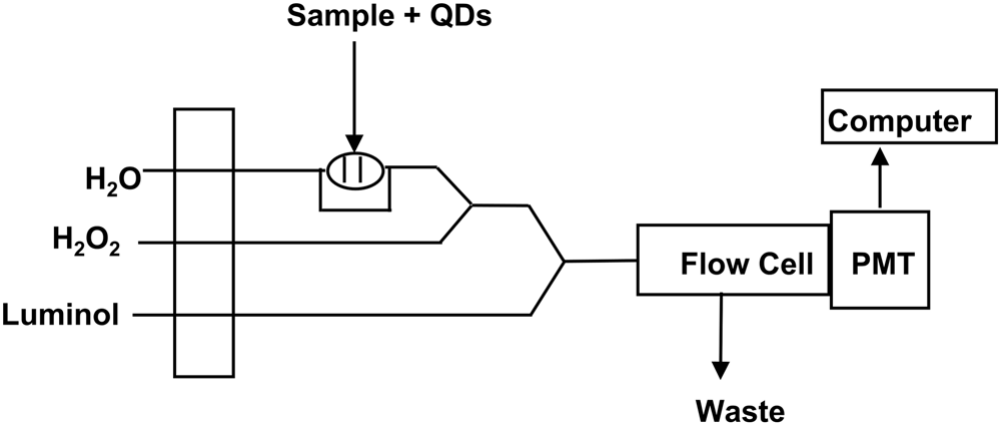
The structure of the flow injection CL system platform. (PMT is photomultiplier tube).

**Figure 2:**
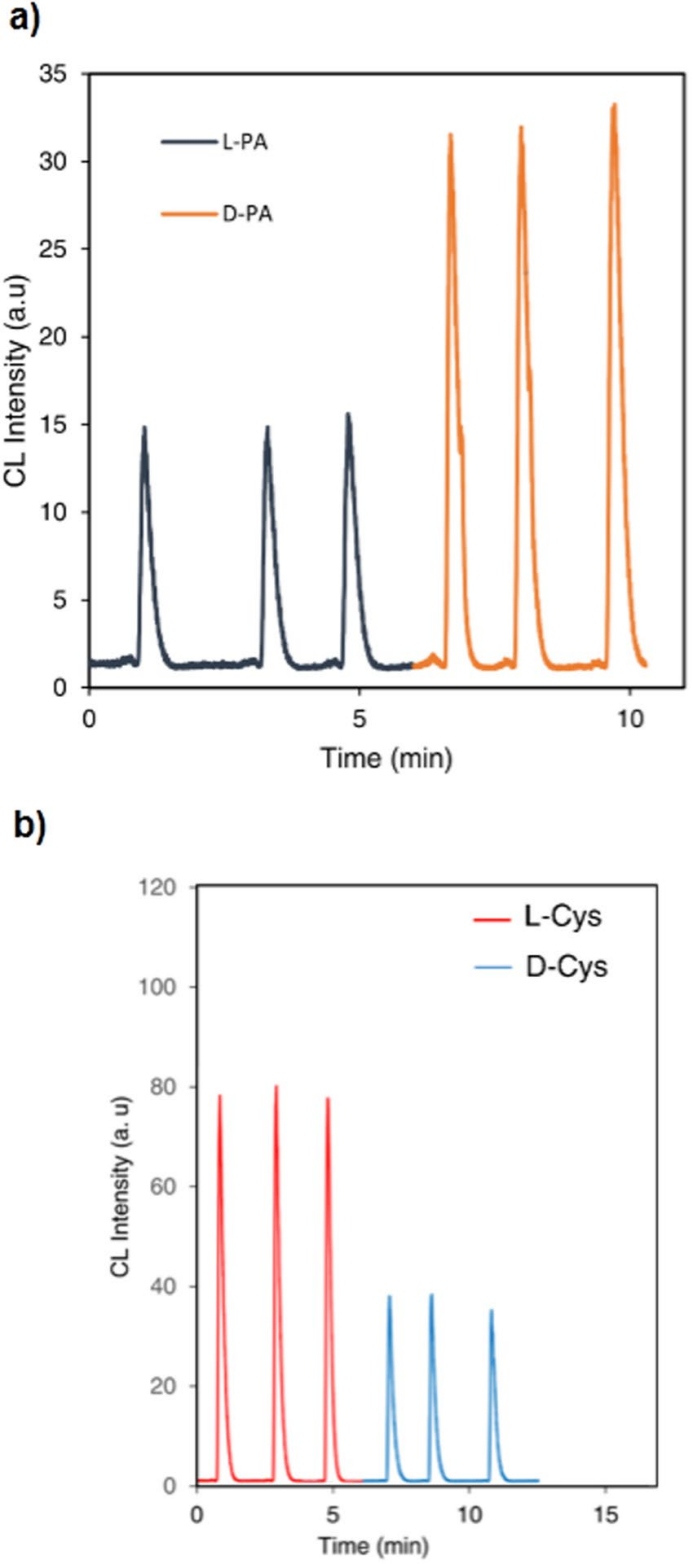
The CL signals of QDs enhanced CL system in presence of enantiomers. (**a**) L-PA and D-PA. (**b**) L-Cys and D-Cys (enantiomers concentration is 2.3 mM).

In the present work, we have demonstrated a novel application of QDs catalyzed CL system for chiral discrimination and determination of enantiomeric excess of D/L- PA and Cys based on the difference in the CL intensities. The circular dichroism (CD) measurement confirmed that L- and D-PA and Cys exhibited mirror-image profiles (see Fig. S1). CL responses of the QDs-luminol-H_2_O_2_ system in the presence of PA enantiomers were investigated. PA enantiomers were added to QDs solution. After the interaction of PA enantiomers with QDs, the mixture solution was injected into the ultrapure water as a carrier stream through a loop-valve injector, mixed with luminol and H_2_O_2_ solutions through three-way pieces and the CL signals were recorded (see Fig. 1). Interestingly the peak intensities of (L-PA, QDs) and (D-PA, QDs) were apparently different. As shown in Fig. 2a, the CL peak intensity of L-PA was much lower than that of D-PA. The distinct signals for the two enantiomers show the QDs-based CL assay is satisfactorily capable of discriminating PA enantiomers. We assume that the differences in observed CL intensities in the presence of PA enantiomers are related to the different interaction of PA enantiomers towards CdTe QDs and also the H_2_O_2_ scavenging effect of PA. Based on the previous reports^60–63^, it is well known that PA has an antioxidant effect and is able to scavenge H_2_O_2_. On the other hand, Zhou *et al*.^64^ reported that D-PA has a stronger interaction with CdTe QDs compared with L-PA. Their calculation results were also illustrated that the energies of D-PA/ CdTe QDs (9239.0 kcal mol^−1^) that is higher than that of L-PA/ CdTe QDs (9232.0 kcal mol^−1^). In addition, our surface-charge density analysis revealed that zeta potentials of the CdTe QDs, L-PA/ CdTe QDs, and D-PA/CdTe QDs were −2.46, −7.96, and −18.1 mV, respectively that shows more interaction of D-PA with CdTe QDs compared with L-PA. Therefore we reasoned that more affinity of D-PA to QDs causes that a higher number of D-PA molecules attach to QDs and less H_2_O_2_ scavenging occurs, leading higher CL intensity compared with L-PA.

**Figure 3:**
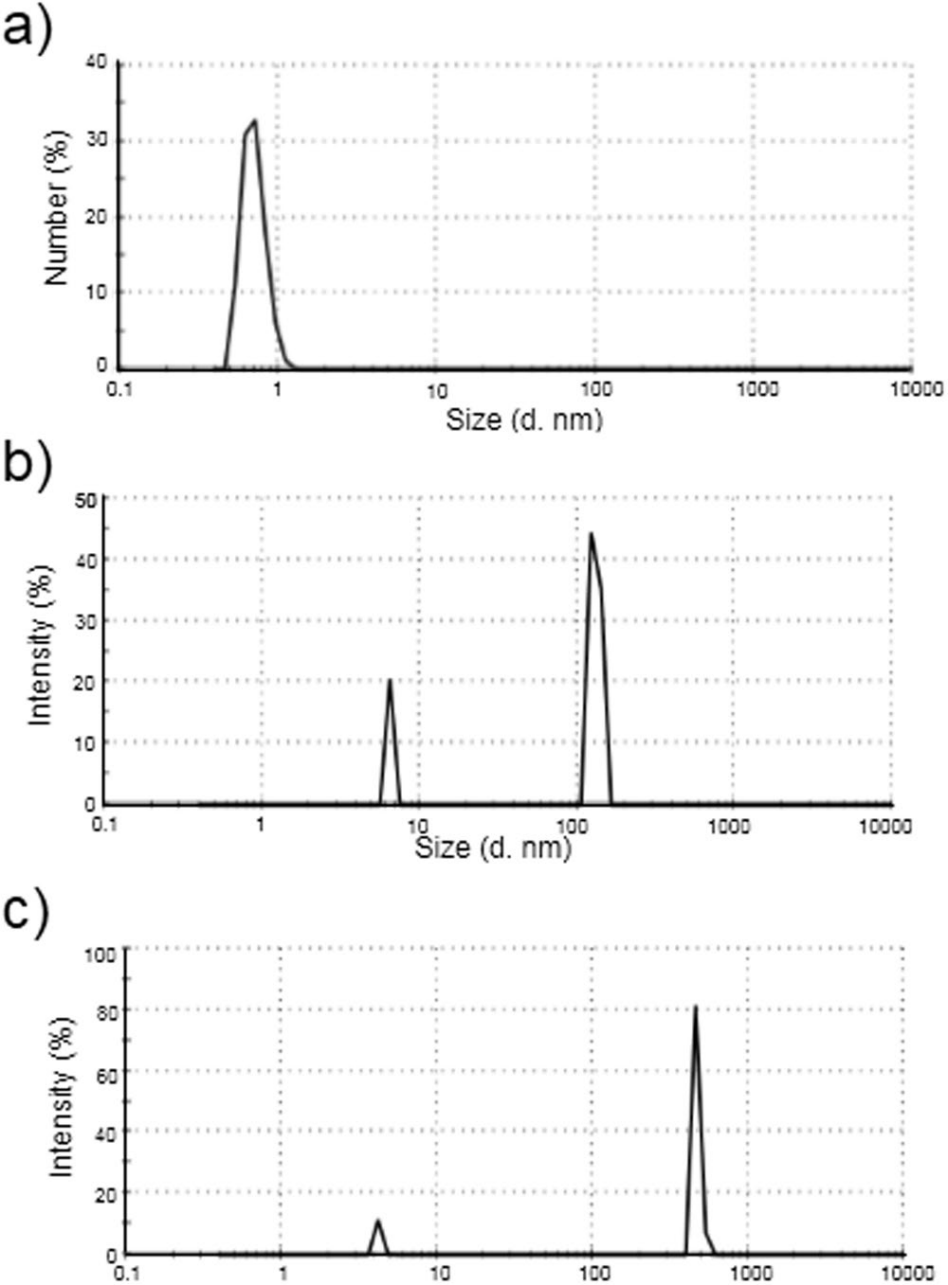
DLS of CdTe QDs. (**a**) Before, and after treatment with 2.3 mM (**b**) L- Cys (**c**) or D- Cys.

The same procedure was done for Cys enantiomers. Upon the interaction of Cys enantiomers with QDs, it was found that the peak intensities of L-Cys/QDs and D-Cys/QDs were obviously different, and showed an excellent enantiomeric discrimination. As shown in Fig. 2b, the CL intensity in the presence of L-Cys was noticeably higher than D-Cys at the same concentration level. Dynamic Light Scattering (DLS) technique was used to investigate the interaction between CdTe QDs and Cys enantiomers. L-Cys and D-Cys were added to QDs solution and the particle size distribution of QDs was recorded in the absence and presence of Cys enantiomers (see Fig. 3). Size change of QDs caused by D-Cys was much larger than that of L-Cys. This should be noted that the difference in the size of QDs in presence of Cys enantiomers was much greater than the difference in presence of PA enantiomers (Fig. S2). As it has reported, D-Cys exhibits a stronger interaction affinity towards CdTe QDs, compared to L-Cys which induces more aggregation^64,65^. On the other hand, it has been widely studied that NPs can enhance luminol–H_2_O_2_ CL intensity by catalyzing the decomposition of H_2_O_2_^50,66^. CdTe QDs have been utilized to significantly catalyze the CL systems such as luminol–H_2_O_2_ CL system^1,67^. The catalytic effect of NPs is related to their sizes and it increases with the available surface area of the catalyst^50^. The surface areas of the NPs decrease with increasing particle size that caused by aggregation and therefore decrease their catalytic efficiencies. Based on the enhancement effect of QDs in luminol-H_2_O_2_ CL system and considering the more aggregation of QDs by D-Cys isomer, it is expected that the less CL intensities be observed as a result of the less exposed surface area of QDs for catalytic effect. The clear difference in the results of the two enantiomers suggests that our simple CdTe QDs-based CL assay is capable of enantiorecognition of PA and Cys. Zhou *et al*.^64^ reported that through the strong interaction between thiol and the Cd atom, cysteine and penicillamine could attach to the CdTe QDs by building Cd-S bonds. On the basis of their empirical observations and calculation results based on the CdTe atomic model, they concluded that D-isomers (Cys or PA) have a stronger interaction with CdTe compared with L-isomers (Cys or PA). We also observed these different affinity interactions via UV-Vis and IR spectrometries (Figs S3, S4).

### Optimization of experimental conditions

**Figure 4:**
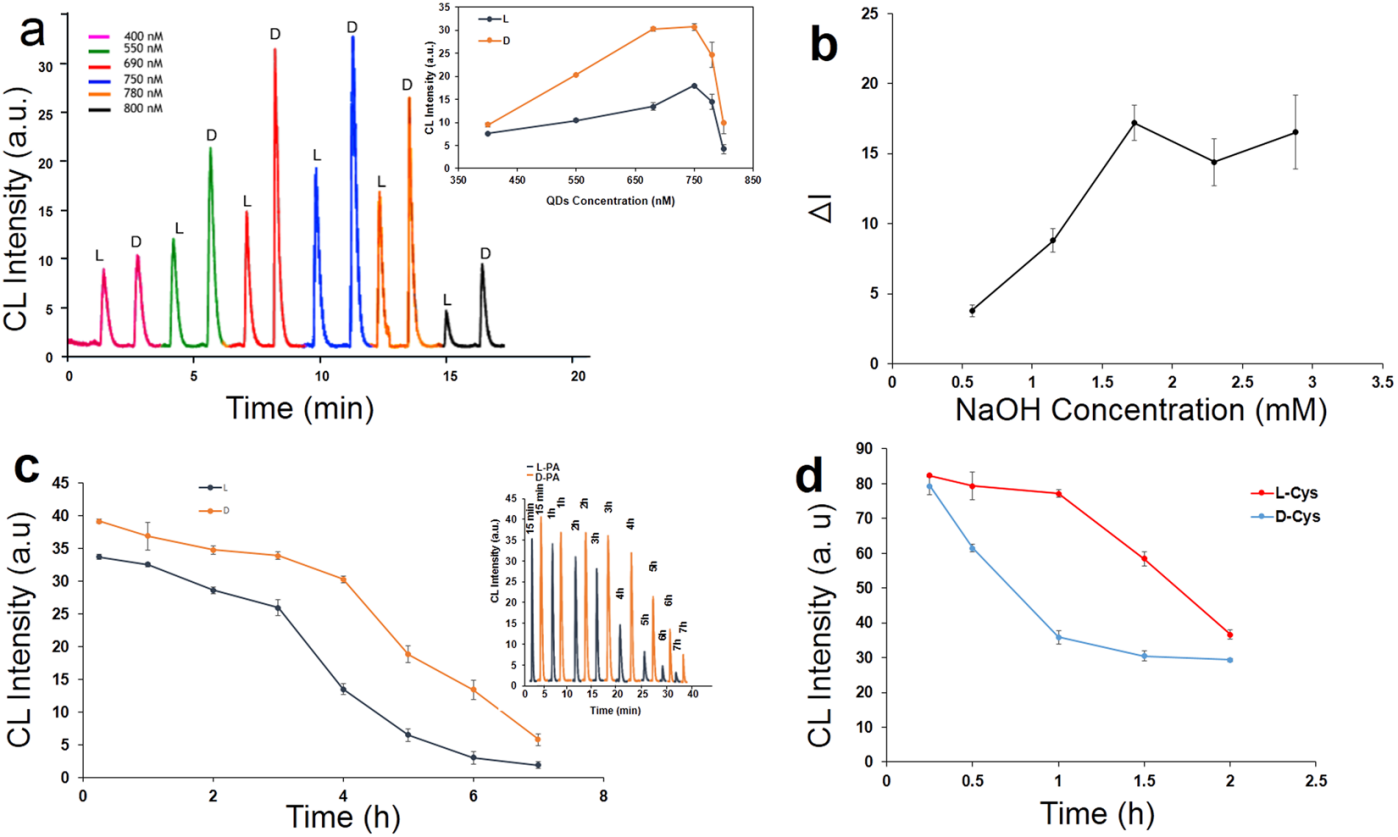
Optimization of experimental conditions. The influence of (**a**) QDs concentration, (**b**) NaOH concentration, (**c**) incubation time on chiral discrimination of PA, and (**d**) incubation time on chiral discrimination of Cys.

The optimal conditions for luminol–H_2_O_2_–NPs CL system including 0.4 mM of luminol, 0.15 M of H_2_O_2_ and 0.01 M of NaOH were utilized according to our previous report^59^. Considering the CL intensity difference in presence of L-PA and D-PA, parameters consisting of the concentration of QDs, the concentration of NaOH and incubation time were tested. As shown in Fig. 4a, a significant role of the concentration of CdTe QDs in the detection and differentiation can be clearly seen. It is obvious that a low response is appeared in a low concentration of QDs because of less catalytic effect. The CL signal decreased in a high concentration of QDs because it can induce more aggregation of QDs. The experimental results showed that the highest ΔI (I-I_0_) for chiral discrimination of L-PA and D-PA was obtained when the QDs concentration was 680 nM (77%) which was chosen as optimum concentration. To obtain the maximum ΔI, the effect of NaOH concentration was also investigated over the range of 0.5 to 2.8 mM. As Fig. 4b shows, the highest difference in CL intensity and thus the maximum chiral recognition possibility appears at 1.7 mM of NaOH which was chosen as the optimum condition to maintain a high sensitivity. The influence of incubation time on the CL response and chiral discrimination was examined in the range of 15 min to 7 h. The changes in peak intensities of L-PA and D-PA with the incubation time are shown in Fig. 4c. The largest difference in peak intensities (ΔI) appeared at 4 h that was selected as the best incubation time for PA measurements.

In order to obtain the best CL intensity difference in presence of L-Cys and D-Cys, the incubation time was investigated. The experimental results showed that the highest ΔI for chiral discrimination of L-Cys and D-Cys was obtained when incubation time was 1 h (see Fig. 4d).

### Analytical performance of method

**Figure 5:**
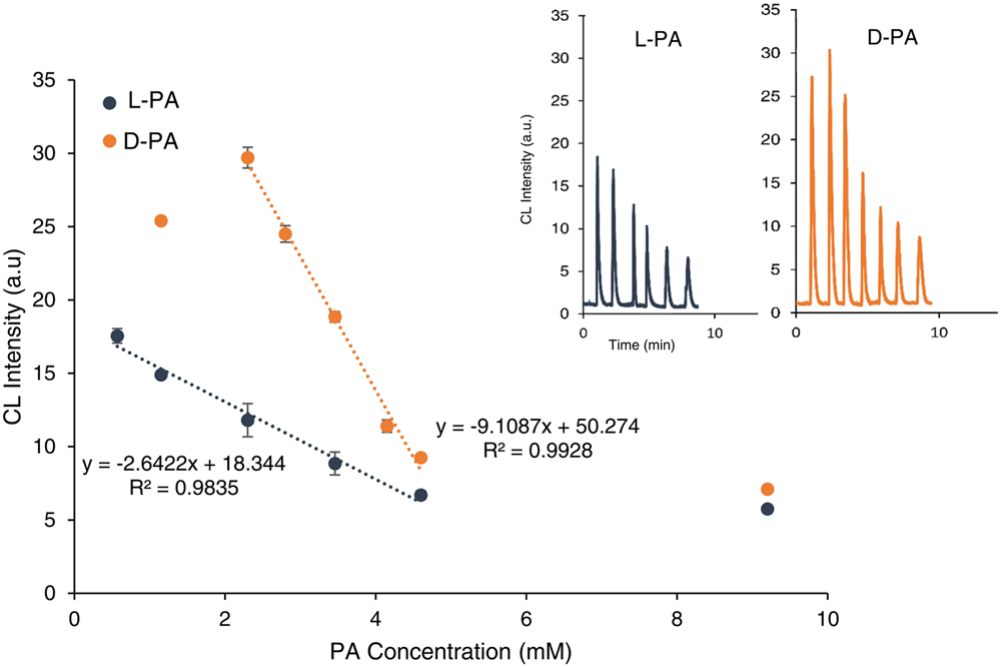
The calibration plots. The CL response in the presence of different concentrations of L-PA and D-PA.

Under optimized conditions, the quantitative range of the proposed method for the detection and discrimination of PA enantiomers was investigated. It was found that the CL signal decreased upon increasing the concentration of PA enantiomers. Higher PA concentration led to more H_2_O_2_ scavenging, which conducted to lower CL intensity. Correspondingly, a linear relationship from 0.5 mM to 4.6 mM, and 2.3 mM to 4.6 mM was obtained for L-PA and D-PA, respectively (see Fig. 5). The results demonstrate that the use of the proposed method allows not only the chiral recognition but also the quantitative analysis of PA. In addition, as shown in Fig. 5, this method presents excellent chiral discrimination performance in a range of concentrations (1.15 mM to 9.2 mM), not only at a single concentration.

**Figure 6:**
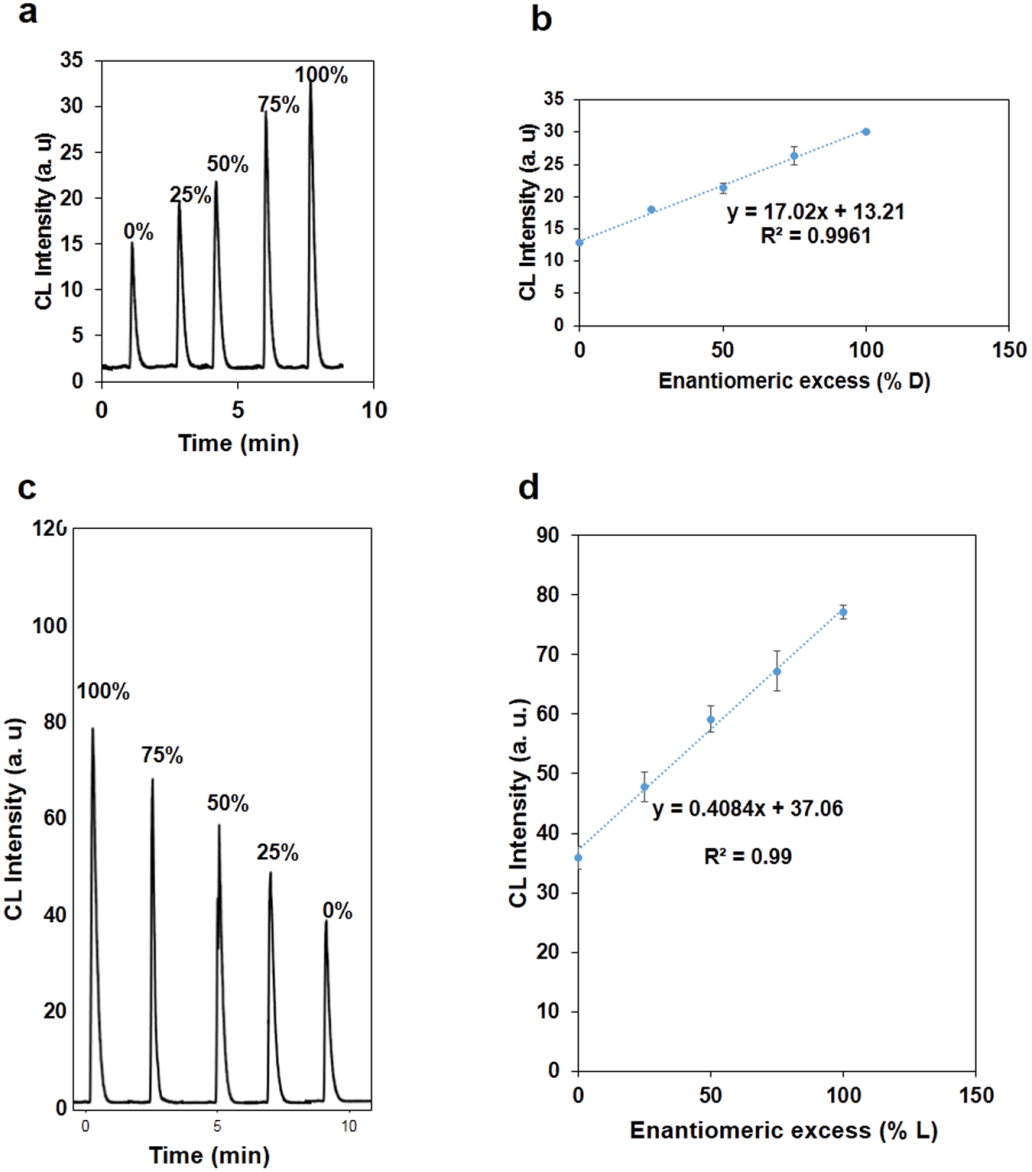
Enantiomeric excess results. (**a**) CL signals in the different enantiomeric excess of PA, (**b**) Plots of CL peak intensity versus the enantiomeric excess of PA, (**c**) CL signals in the different enantiomeric excess of Cys, (**d**) Plots of CL peak intensity versus the enantiomeric excess of Cys (the total concentration of D- and L-enantiomers is 2.3 mM).

The enantiomeric excess (ee) means the absolute difference between the mole fractions of each enantiomer. The enantiomeric excess determination is necessary for many fields, such as asymmetric catalyst discovery and chiral drug production^1^. Therefore, this system was applied to determine the enantiomeric excess of PA and Cys. A series of enantiomeric compositions (L/D = 100/0, 75/25, 50/50, 25/75, 0/100) of PA and Cys were prepared and their effect on the CL of CdTe QDs-CL system was studied. The relationship between the enantiomeric excess of targets and CL was obtained by keeping the total concentration of two isomers the same (*i.e*. 2.3 mM) for this assay system. As shown in Fig. 6, the intensity of CL gradually changes with increasing D- or L-isomer proportion. The observed CL intensities were linearly proportional to the enantiomeric excess of D- or L-isomer. Thus, the enantiomeric composition of PA and Cys can be quantified using analysis of the CL responses in this system.

**Figure 7:**
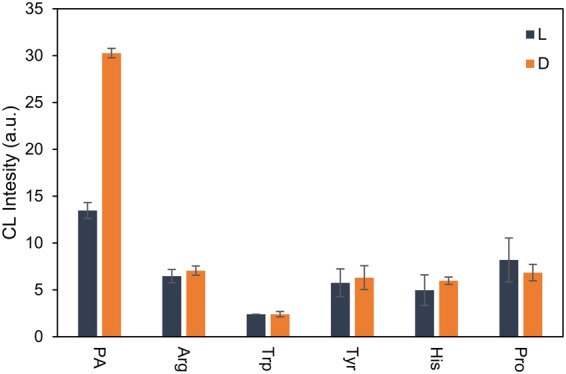
Selectivity evaluation towards interferences on the optimized conditions of PA discrimination. (The concentration of all compounds is 2.3 mM).

Selectivity of the method was evaluated by investigation of the response of the QDs-luminol-H_2_O_2_ CL system towards other amino acids at the same concentration, including arginine, tryptophan, tyrosine, proline, and histidine. As shown in Figs 7 and S5, at the same condition, the signal of QDs-luminol-H_2_O_2_ adding D-enantiomer was almost the same as L-enantiomer. The results indicated that proposed method has high selectivity over other amino acids enantiomers under our experimental condition. Since cysteine and penicillamine could attach to the CdTe QDs by building Cd-S bonds and differences in these interactions led to different CL intensities, the proposed method has high selectivity over other amino acids enantiomers that are lack of thiol groups.

### Real sample Analysis

In order to assess the application of the proposed method for the detection of PA in real samples, the designed approach was applied to determine the concentration of D-PA in tablet and urine samples spiked by D-PA or L-PA. The results in Table 1 demonstrate satisfying recovery values (between 92.50–103.04%) and low relative standard deviations (RSD% between 2.30–3.66%).

**Table 1:** Measurement of D-PA and L-PA in urine and tablet samples.

Sample	Added PA (mM)	Found PA (mM)	Recovery(%)	RSD (%)
Urine	0	0	—	—
Urine	2.30 of L-PA	2.19	95.2	3.66
Urine	2.30 of D-PA	2.37	103.04	2.30
Tablet	0	4.04	—	2.50
Tablet	0.40 of D-PA	4.43	92.50	2.63

## Conclusion

In this work, a simple and novel strategy was utilized for chiral recognition of PA and Cys using QDs enhanced-CL system. This effective enantioselective sensing method was based on an obvious difference in the CL intensities in presence of L and D-enantiomers. The proposed chemiluminometric method demonstrated excellent performance to determine the enantiomeric excess (ee) of PA and Cys in the whole range of ee values. Moreover, the proposed method exhibited good linearity for PA quantification. Practical detection of PA in pharmaceutical tablet and urine sample was also successfully examined. In comparison with conventional analysis for chiral discrimination such as HPLC, GC, CD, and NMR, this technique has many advantages because of its simple instrumentation, low cost, and high selectivity. We believe that NP enhanced-CL systems can find important applications, including their use as chiral recognition sensors.

## Methods

### Materials and reagents

Thioglycolic acid (TGA, 99%), tellurium powder (Te, 99.8%), and cadmium chloride (CdCl_2_. 2H_2_O) were obtained from Sigma. Sodium borohydride (NaBH_4_, 98%), 5-amino-1,2,3,4-tetrahydro-1,4-phthalazinedione (luminol, 95%), hydrogen peroxide (H_2_O_2_, 30%), and sodium hydroxide (NaOH) were bought from Merck. Penicillamine (PA), cysteine (Cys), arginine (Arg), tryptophan (Trp), tyrosine (Tyr), proline (Pro), and histidine (His) enantiomers were purchased from Sigma. Milli-Q grade water (18.2 MΩ.cm at 25 °C) was used throughout the experiments.

### Apparatus

Circular dichroism (CD) measurements were recorded on a Jasco J-810 CD spectropolarimeter. Size distributions were obtained from Dynamic Light Scattering (DLS) by using Zetasizer Viscotec 802 at ambient temperature. UV-Vis spectra were measured and recorded with a Lambda (Perkin Elmer, USA) spectrophotometer with the use of 1.0 cm cell. FTIR spectra of samples were taken using an ABB Bomem MB-100 FTIR spectrophotometer. All the spectra were obtained at room temperature.

### Synthesis of TGA functionalized CdTe QDs

TGA-capped CdTe QDs were synthesized according to the procedure described previously^68^. Typically, 0.256 g of CdCl_2_·5H_2_O and 200.0 µL TGA were added in 4.0 mL ultrapure water. Meanwhile, 3.0 M NaOH was used to adjust the pH to 9.0. In the next step, 0.065 g of Te powder and 0.183 g of NaBH_4_ were added into 75.0 mL ultrapure water under argon flow at 50 °C in a three neck round bottom flask. The solution was refluxed with vigorous stirring until the solution turned purple. Subsequently, the temperature of the solution was increased to 150 °C and the prepared Cd solution was added to the purple solution. After 2 h of reaction, TGA-CdTe QDs solution was obtained. Finally, the resulting solution was stored in the refrigerator (4 °C). The as-prepared CdTe QDs were characterized by atomic force microscopy (AFM). The size of CdTe QDs was determined by AFM (Fig. S6), and the average height for CdTe QDs was about 6.8 nm.

### Chiral discrimination of PA and Cys enantiomers

D-PA or L-PA was dissolved in water containing 150 μL of 0.5 M of NaOH to a total 10 mL volumetric flask to obtain different concentrations of PA. 466 µL of the synthesized TGA-CdTe QDs were diluted with DI water to a final volume of 25 mL. In a 2 mL eppendorf tube, 1 mL of diluted QDs solution and 300 μL D-PA or L-PA solution at different concentrations were added, and then the mixture was incubated for 4 h at room temperature. For flow-injection chemiluminometric chiral discrimination of D-PA and L-PA, a laboratory-built flow injection CL system was used. As shown in Fig. 1, the mixture of PA and QDs was injected into the water as a carrier through a 200 µL loop-valve injector, mixed with H_2_O_2_ and luminol solutions through three-way pieces. Then, the mixed solution moved to a flow cell with spiral-shape which was located in front of a photomultiplier tube (PMT) and the CL signal was then monitored. The same procedure was done for L-Cys and D-Cys except that it was incubated for 1 h at room temperature. The process of enantiomeric excess detection was the same as that recognition of D- and L-enantiomers, upon addition of 300 μL Cys or PA with the different enantiomeric excess solution to 1 mL of QDs.

### Application to real samples

The practical application of the proposed method was evaluated by using pharmaceutical tablets and urine samples. A fresh urine specimen was prepared from a healthy volunteer and a fifty-fold sample dilution was made. To assess the recovery and precision D-PA and L-PA were spiked into the urine sample under defined optimal conditions prior to CL measurement. A pharmaceutical tablet was dissolved in 50 mL pure water and was filtered to remove large insoluble particles. The clear solution was then subjected to the same CL assay under optimized conditions. This study was carried out in accordance with relevant guidelines and regulations and has been approved by the ethical committee of the Sharif University of Technology. Informed consent was obtained from all subjects.

## Electronic supplementary material

Supporting Information
