# Supplementary material for: Nanoparticle-based Chemiluminescence for Chiral Discrimination of Thiol-Containing Amino Acids
Source: Sci Rep. 2018 Sep 18;8:14011. doi: 10.1038/s41598-018-32416-z (PMC6143635; doi:10.1038/s41598-018-32416-z)
Supplement: Supplementary file 1 — Supporting Information [file 41598_2018_32416_MOESM1_ESM.doc]

**Supporting Information**

**Nanoparticle-based Chemiluminescence for Chiral Discrimination of Thiol-Containing Amino Acids**

**Maryam Shahrajabian^1^, Forough Ghasemi^1^, M. Reza Hormozi-Nezhad^1, 2*^**

^1^Department of Chemistry, Sharif University of Technology, Tehran, 11155-9516, Iran

^2^Institute for Nanoscience and Nanotechnology, Sharif University of Technology, Tehran, Iran

*Email: [hormozi@sharif.edu](mailto:hormozi@sharif.edu)

**(a)**


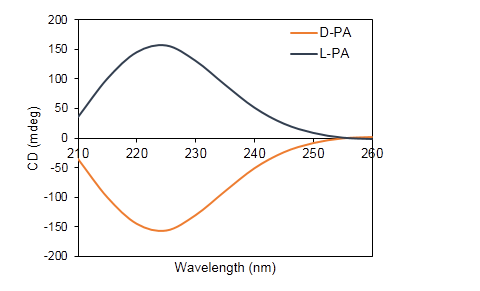


**(b)**

**
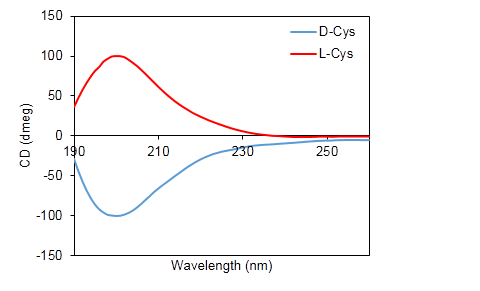
**

**Figure S1. The CD spectra**. **(a)** L-PA and D-PA. **(b)** L-Cys and D-Cys.


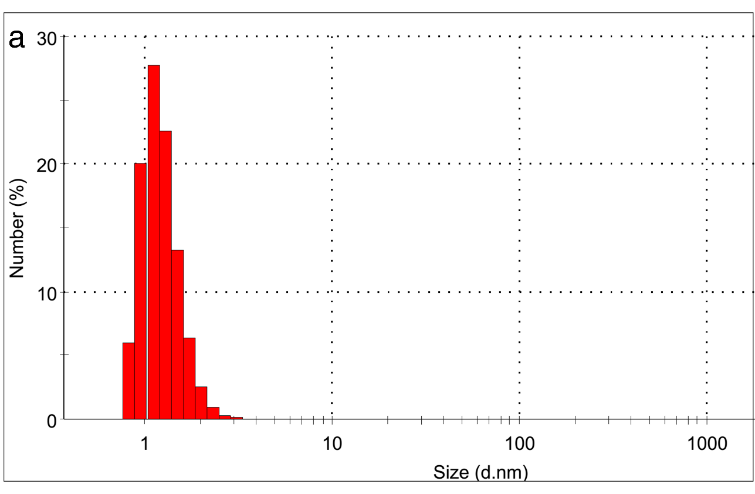


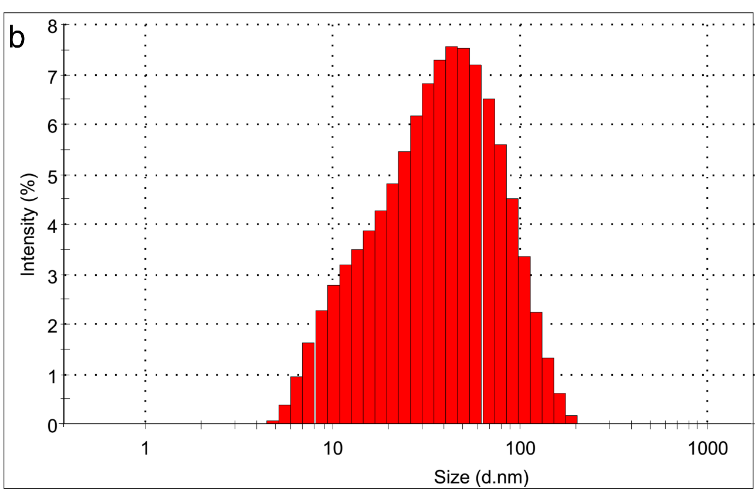


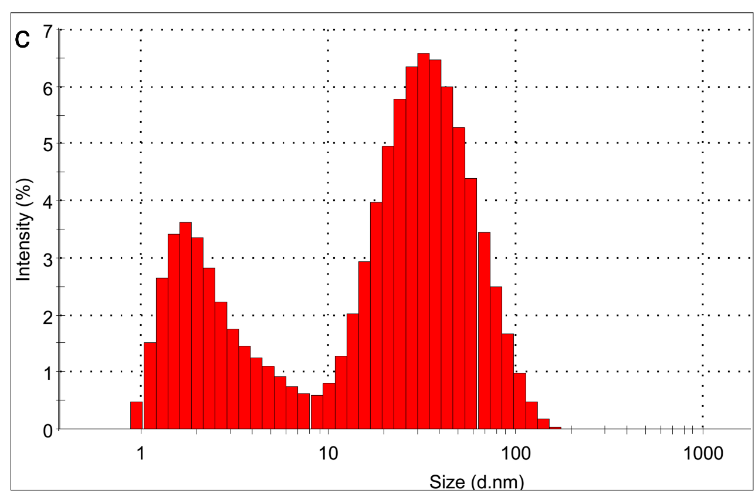


**Figure S2. DLS of CdTe QDs**. **(a**) Before, and after treatment with 2.3 mM of (**b**) L-PA or (**c**) D-PA.


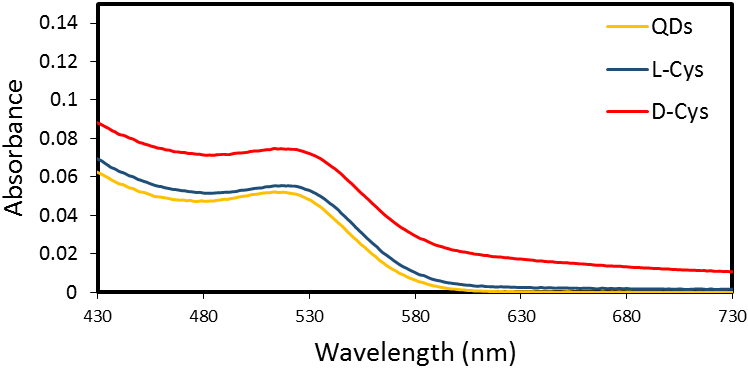


**Figure S3.** **The UV-Vis spectra**. Absorption spectra of CdTe QDs in the presence of 2.3 mM of L-Cys or D-Cys. More interaction of D-isomer could be observed. More interaction leads to more aggregation and bigger size of QDs caused more scattering effect and bigger absorbance intensity.

**
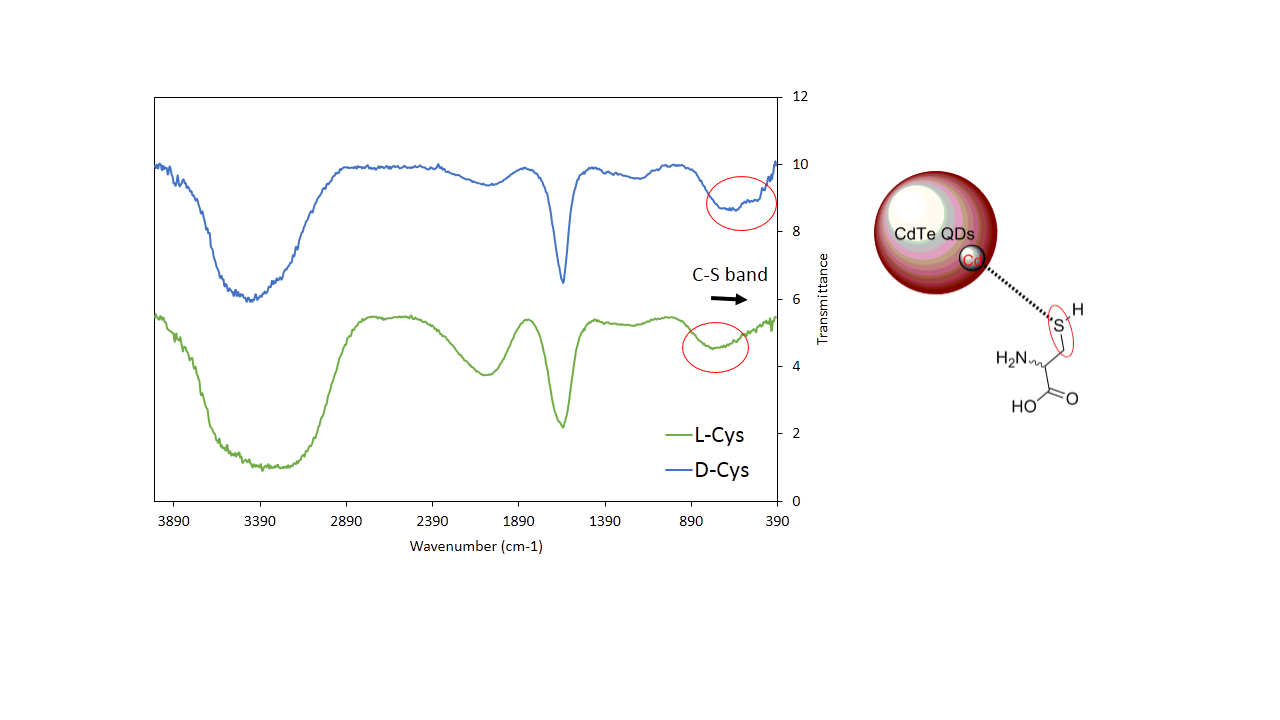
**

**Figure S4**. **The FTIR spectra.** FTIR spectra of CdTe QDs in the presence of 2.3 mM of L-Cys or D-Cys. More interaction of D-isomer could be observed. Stronger interaction between thiol and the Cd atom leads to sift of the C-S band to lower wavenumber.

**
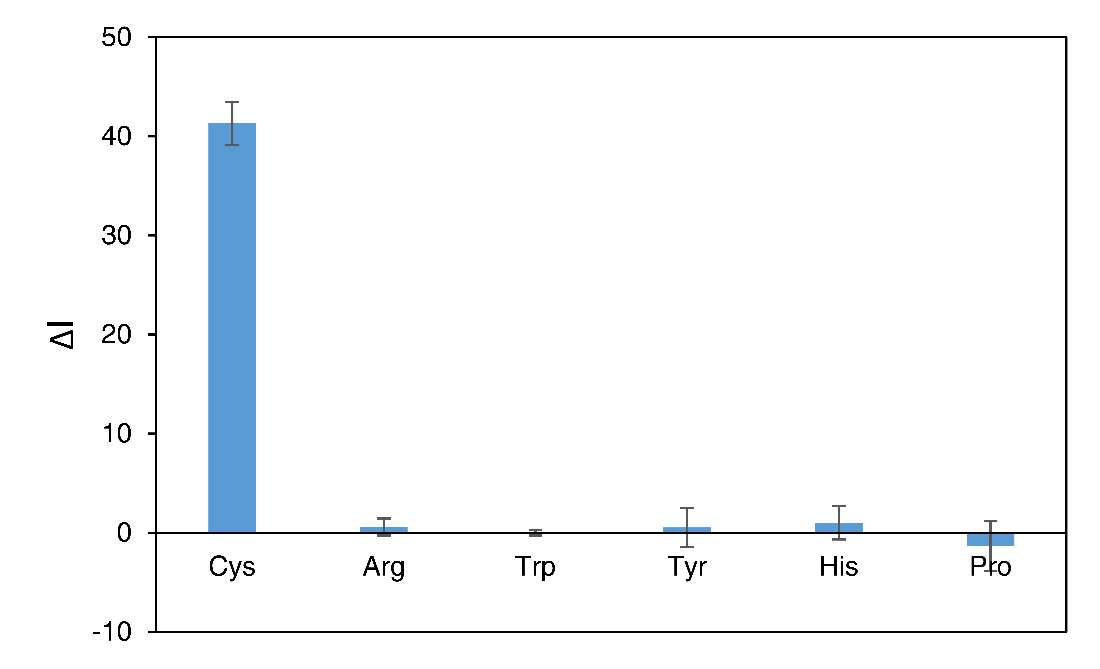
**

**Figure S5. Selectivity evaluation towards interferences on the optimized conditions of Cys discrimination**. (the concentration of all compounds is 2.3 mM).

**
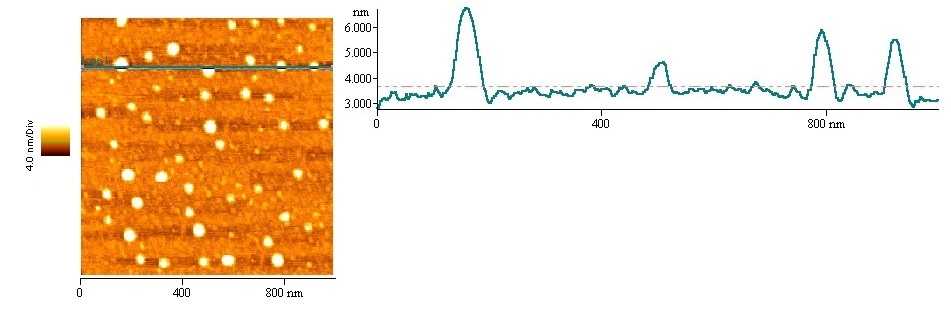
**

**Figure S6. AFM of CdTe QDs.**
